# “How Much Will I Get Charged for This?” Patient Charges for Top Ten Diagnoses in the Emergency Department

**DOI:** 10.1371/journal.pone.0055491

**Published:** 2013-02-27

**Authors:** Nolan Caldwell, Tanja Srebotnjak, Tiffany Wang, Renee Hsia

**Affiliations:** 1 Department of Emergency Medicine, Stanford University, Stanford, California, United States of America; 2 Ecologic Institute, San Mateo, California, United States of America; 3 University of Minnesota Medical School, Twin Cities, Minnesota, United States of America; 4 Department of Emergency Medicine, University of California San Francisco, San Francisco, California, United States of America; Old Dominion University, United States of America

## Abstract

**Objectives:**

We examined the charges, their variability, and respective payer group for diagnosis and treatment of the ten most common outpatient conditions presenting to the Emergency department (ED).

**Methods:**

We conducted a cross-sectional study of the 2006–2008 Medical Expenditure Panel Survey. Analysis was limited to outpatient visits with non-elderly, adult (years 18–64) patients with a single discharge diagnosis.

**Results:**

We studied 8,303 ED encounters, representing 76.6 million visits. Median charges ranged from $740 (95% CI $651–$817) for an upper respiratory infection to $3437 (95% CI $2917–$3877) for a kidney stone. The median charge for all ten outpatient conditions in the ED was $1233 (95% CI $1199– $1268), with a high degree of charge variability. All diagnoses had an interquartile range (IQR) greater than $800 with 60% of IQRs greater than $1550.

**Conclusion:**

Emergency department charges for common conditions are expensive with high charge variability. Greater acute care charge transparency will at least allow patients and providers to be aware of the emergency department charges patients may face in the current health care system.

## Introduction

Emergency Departments (EDs) play a key role in the delivery of health care services for a wide variety of acute medical needs. [1] One in every five Americans has at least one visit to the ED per year. [2] Although many people depend on the ED, obtaining acute medical care is increasingly becoming a significant financial burden as total charges for ED services continue to rise. [3] To the consumers with insurance coverage, these growing charges result in larger deductibles and co-payments as payers shift toward increased cost sharing. [4] To the growing uninsured who particularly rely on the ED, elevated charges directly result in higher proportions of self-pay responsibility. [5], [6] Regardless of insurance status, increasing charges are growing difficult to manage as aggregate out-of-pocket payments for healthcare have been projected to continue their growth and double from 3.0% to 6.0% per year between 2010–2019. [7] In fact, financial concerns have been cited as the number one reason individuals with non-urgent medical issues delay treatment until an urgent/emergent condition develops. [8].

Rising healthcare charges and associated system cost control have been at the forefront of recent economic, political, and medical discussion. [9] Medical charge transparency has been touted as necessary to create market competition that narrows price ranges and lowers overall consumer cost. [10], [11] There have been some efforts to increase charge transparency by creating price indexes for many inpatient procedures and medical care. [12] However, similar efforts to inform consumers of expected charges for common outpatient ED treatments have been lacking due to the unavailability of this data in most administrative datasets. Patients in the ED are still uninformed, underestimate their financial responsibility, and are often shocked at the charge posted on their bill. [13], [14] The majority of providers are similarly inaccurate when asked by patients regarding billable charges of their visit. [15] To our knowledge, no study has yet shown the wide range of charges for outpatient treatment for common conditions in the ED. Therefore, in an effort to inform physicians and consumers, we seek to describe patient charges and their variability for diagnosis and treatment of the ten most common outpatient conditions presenting to the emergency department from 2006–2008.

## Methods

### Study Design and Setting

This is a cross-sectional study of the 2006–2008 Medical Expenditure Panel Survey (MEPS), a public data source from the US Agency for Healthcare Research and Quality. The MEPS uses a complex sampling design based on the National Health Interview survey framework, and then applies survey weights to the absolute results to create representative estimates of the United States medical diseases profile, patient demographics, healthcare utilization and charges. [16] The sampling weights are designed to account for differential non-response rates and oversampling of underrepresented groups, among other factors. [17], [18].

The MEPS uses multiple panels of households to create an overlapping data set from year to year. Each household serves for two full years on a panel, with the last year of data used to create overlap with new households on the panel. The designated household representative submits information for each individual household member. The MEPS collects information from the respondent on inpatient and outpatient medical usage from patient interviews and medical diaries. This data is then cross-referenced with provider and insurer records to ensure validity.

Our study was exempt from the Institutional Review Board at the University of California, San Francisco because we used a public data source that was masked for identifiers.

### Data Collection and Processing

We gathered data on ED use along with associated ED charges from the 2006–2008 MEPS. We merged two MEPS data files, one with ED visits and the other with population characteristics, using a unique patient identifier. Patient demographics, insurance status and medical comorbidities were gathered from the MEPS population characteristics file, while the clinical characteristics for each visit were taken from the ED event file.

For each ED patient encounter, MEPS reports up to three 3-digit International Classification of Disease, Ninth Revision (ICD-9) condition codes along with an associated Clinical Classification Software (CCS) code (Agency for Healthcare Research and Quality, Washington, DC). The CCS condition code was used as the listed encounter diagnosis for our analysis.

### Patient Selection

**Figure 1:**
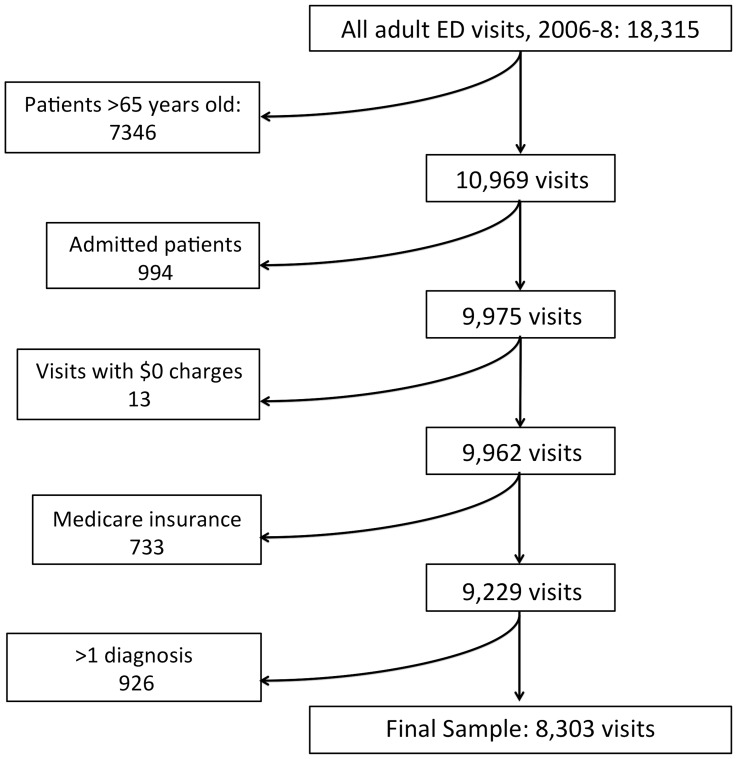
Sample selection process.

Each ED encounter in MEPS between 2006 and 2008 was used as a separate unit of analysis regardless of patient identifier (n = 18,315). Our patient selection process is outlined in Figure 1. We excluded encounters with elderly patients >65 years of age (n = 7346) because the majority of them are covered by Medicare, and we wanted to focus on adults age 18–64 who are at the highest risk of facing the largest out-of-pocket charges on their bills. [19] We further chose to focus on outpatient conditions and therefore excluded all visits resulting in admission (n = 994). ED encounters with multiple listed discharge diagnoses were also excluded (n = 926) to try and create a simple, more homogeneous sample for each diagnosis by removing outpatient ED visits complicated by other conditions. We excluded several entries (n = 13) that had an ED charge of zero as these were assumed to be data errors or otherwise not suitable for analysis with our outcome of interest. The final, unweighted sample size of patients we deemed eligible for the study was 8,303.

### Outcome Measure

Our primary outcome measure was total charge. The total charge recorded in the MEPS includes the sum of medical care, tests, and treatment (facility and physician fee). These charges do not represent the amount patients or insurers reimburse providers, but rather the total charge that patients or their insurance providers are billed.

### Data Analysis

We began by analyzing the demographic breakdown of the absolute and weighted number of visits (Table 1). We then generated the top ten diagnoses by totaling all ED visits during our study period and ranking them in order of frequency by primary diagnosis. We then examined charges for each diagnosis, and within diagnoses we looked at median charges by insurance provider (Medicaid, private, or uninsured). We chose to present the median charge for these ED visits to prevent outliers in each disease category skewing the interpretation of the charges central tendency. We used simple descriptive statistics to determine differences between and among insurance groups as well as conditions. All charges were indexed to levels of 2008 dollars using the US Consumer Price Index. All analyses were performed with R (Version 2.10.1 2009-12-14, The R Foundation for Statistical Computing).

**Table 1:** Patient demographics.

Characteristic	Observations(n = 8,303)	Weighted visits (In millions)(n = 76.6 million)
Age:		
18–24	1556 (18.7%)	13.9 (18.2%)
25–29	1110 (13.4%)	10.5 (13.8%)
30–39	1892 (22.8%)	16.3 (21.4%)
40–49	1703 (20.5%)	16.0 (21.0%)
50–59	1545 (18.6%)	14.5 (18.9%)
60–64	497 (6.0%)	5.2 (6.8%)
Sex:		
Male	3089 (37.2%)	31.5 (41.0%)
Female	5214 (62.8%)	45.2 (59.0%)
Insurance:		
Private	4068 (48.9%)	44.7 (58.4%)
Medicaid	1822 (21.9%)	12.7 (16.5%)
Uninsured	3048 (36.7%)	24.7 (32.2%)
Race:		
Asian/Pacific Islander	166 (2.0%)	1.4 (1.8%)
Black	1899 (22.9%)	12.5 (16.3%)
Non White Hispanic	1778 (21.4%)	9.6 (12.6%)
White	4196 (50.5%)	50.9 (66.4%)
Other/multiple race	264 (3.2%)	2.3 (3.0%)
Medical Comorbidities:		
Hypertension	2429 (29.3%)	21.3 (27.8%)
Hypercholesterolemia	1886 (22.7%)	18.2 (23.7%)
Asthma	1481 (17.8%)	13.3 (17.3%)
Diabetes	786 (9.5%)	6.0 (7.8%)

## Results

Our sample consisted of 8,303 observations, representing 76.6 million ED visits. About 22.8% of the observations were from patients between 30–39 years old. Of ED visits, 62.8% were female patients, 48.9% privately insured, and 50.5% self-reported as white. As shown in Table 1, the most common medical comorbidities were hypertension (29.3%), hypercholesterolemia (22.7%) and asthma (17.8%). The most common outpatient conditions were “sprains and strains”, “other injuries”, and “open wounds of extremities,” comprising 5.0%, 4.3%, and 3.8% of total visits, respectively (Table 2).

**Table 2:** Ten most frequent treat and release ED Diagnoses, 2006–2008.

Disease	Observations(n = 2,717)	Weighted Visits(n = 25.3 million)	Rank (Observations, Weighted Visits)
Sprains and strains	415 (15.3%)	4.4 million (17.4%)	1, 1
Other injury	354 (13.0%)	3.3 million (13.0%)	2, 3
Open wounds of extremities	312 (11.5%)	3.4 million (13.4%)	3, 2
Pregnancy	298 (11.0%)	2.1 million (8.3%)	4, 6
Headache	287 (10.6%)	3.1 million (6.7%)	5, 4
Back problems	250 (9.2%)	2.1 million (7.9%)	6, 5
Upper respiratory infection	215 (7.9%)	1.7 million (5.9%)	7, 8
Kidney stone	204 (7.5%)	2.0 million (7.9%)	8, 7
Urinary tract infection	192 (7.1%)	1.5 million (5.9%)	9, 10
Intestinal infection	190 (7.0%)	1.7 million (6.7%)	10, 9

During our study period, the median charge for outpatient conditions in the emergency room was $1233 (95% CI: $1199– $1268). Upper respiratory infections had the lowest median charge of $740 (95% CI: $651– $817), while a urinary tract calculus (kidney stone) was charged the highest median price of $3437 (95% CI: $2917– $3877).

Regarding variability of charges, all diagnoses had an interquartile range (IQR) greater than $800 with 60% of IQRs greater than $1550. The diagnoses with the largest IQRs were: “calculus of urinary tract” (kidney stone) ($3742), “normal pregnancy and delivery” ($2008), and “urinary tract infection” ($1975). Table 3 shows the breakdown of charges for all top ten diagnoses.

**Table 3:** Emergency department charges for the ten most common outpatient conditions.

Diagnosis	Median charge ($) (95% CI)	Mean charge ($) (95% CI)	Inter-quartile range (IQR)	Minimum charge	Maximum Charge
Sprains & strains	1051 (982–1110)	1498 (1304–1692)	1018	4	24110
Other injury	1151 (1003–1281)	2103 (1770–2437)	1594	46	27238
Open wounds of extremities	979 (864–1090)	1650 (1341–1959)	924	29	25863
Normal pregnancy and/or delivery	1204 (1027–1384)	2008 (1701–2315)	2008	19	18320
Headache	1210 (1093–1344)	1727 (1510–1943)	1572	15	17797
Back problems	871 (741–984)	1476 (1265–1687)	1189	66	10403
Upper respiratory infection	740 (651–817)	1101 (891–1312)	827	19	17421
Kidney stone	3437 (2917–3877)	4247 (3642–4852)	3742	128	39408
Urinary tract infection	1312 (1025–1580)	2598 (1780–3416)	1975	50	73002
Intestinal infection	1354 (1114–1524)	2398 (1870–2927)	1960	29	29551
Total outpatient conditions	1233 (1199–1268)	2168 (2103–2233)	1957	3.5	73,002

All diagnoses have an IQR of greater than $800. The diagnoses with the largest IQRs were kidney stone ($3742), normal pregnancy and delivery ($2008), and urinary tract infection (UTI) ($1975).

**Figure 2:**
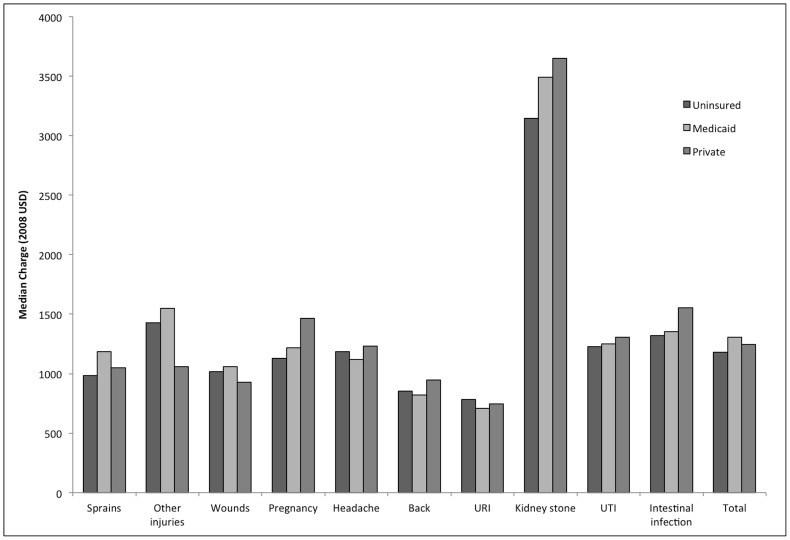
Emergency department charges across payer group for the ten most frequent outpatient conditions. Vertical bars indicate median charge for each of the ten conditions by insurance type: uninsured (black), Medicaid (dark grey), and private insurance (light grey). Medicaid patients were charged the most overall (median $1305), followed by private insurance ($1245), and uninsured patients ($1178).

Analysis by insurance group (Figure 2) was conducted for the aggregate charges of all top ten conditions. Uninsured patients were charged the lowest median price ($1178; 95% CI: $1117–$1241), followed by private insurance ($1245; 95% CI: $1206–$1248) and Medicaid ($1305; 95% CI: $1215–$1395).

## Discussion

### Comment

Using the MEPS 2006–2008, we find previously undocumented patterns in emergency department charges for the ten most frequent outpatient diagnoses. The most frequent outpatient diagnoses were sprains, other injuries and open wounds of extremities. The median charge for outpatient conditions in the emergency department was $1233, which is 40% more than the average American pays in rent each month ($871). [20] Median charges ranged from $740 for an upper respiratory infection to $3437 for a kidney stone. All diagnoses had a high degree of charge variation with 60% of interquartile ranges greater than $1550. Analysis of charges across insurance groups for outpatient emergency department visits revealed that the uninsured have the lowest median charges followed by private insurance, and Medicaid.

While overall in hospital charge burden and variation by diagnosis has been well studied, [21] our analysis is the first to our knowledge to demonstrate the large nationwide charge variability in common emergency department procedures not resulting in admission. [22] Our study is not designed to evaluate the specific reasons behind the large charge variations we observed; previously cited causes for in-hospital care variation include clinical severity and between hospital differences due to factors including geographical differences, provider reimbursement variation, and health care monopolies. [23], [24], [25] However, our analysis indicates for the first time that these and likely other inefficiencies exist in the acute care system and lead to unpredictability and wide variability of healthcare costs for patients. These inefficiencies, if addressed, could help control healthcare costs in the emergency department.

We report ED charges as they would appear on a patient’s bill, revealing the discrepancies in charges for the same diagnoses that patients are generally unaware of. Providers are often at a loss when their patient questions them about the charges for a certain procedure or treatment. [26] Even less common knowledge is how charges at the patient’s current hospital compare to others. All too often a patient presents to the ED with the reasonable question, “How much will I get charged for this?” and providers are unable to answer. Though clearly we cannot expect providers to predict the resource utilization required for an undifferentiated patient, we believe that they should at least be more informed of the variability of charges in our current system.

It is important to note that while these ten most common conditions comprised 32% of outpatient ED visits, these are most likely not the most costly conditions. Had we chosen a methodology to isolate the most costly conditions, the median and mean charges would be much higher. Our goal was to provide a representation of the burden of the most common conditions, rather than the most expensive conditions.

Efforts to increase price transparency have been proposed by over 30 states and are being pursued by the public and private sector as the next phase in medical care. [27] Charge transparency could help patients make more informed, cost-effective personal decisions about their emergency department care. While most patients with time-sensitive conditions such as acute myocardial infarction, stroke, or sepsis may not be in a position to make decisions about their care based on costs or charges, there are many situations in which patients could reasonably inquire about the potential financial implications of their medical care before making treatment decisions. In the current system the financial consequences of medical care can be significant; of non-elderly Americans, 41% report outstanding medical debt and 60% of US bankruptcies are attributable to unpaid medical bills. [28] With the Affordable Care Act due to expand coverage significantly in coming years, especially to Medicaid patients who disproportionately rely on the ED for care, [1] cost control will be come even more important. Charge transparency warrants further investigation in the ED, especially for less time-sensitive conditions, as cost-control measure that can also increase patient self-efficacy.

Further research should examine the sources of this variation in care within diagnoses in the emergency department, as well as how charge transparency could work to reduce this variability and increase healthcare efficiency.

### Limitations

Our study has several limitations given its retrospective design and information available for analysis in the Medical Expenditure Panel Survey. First, MEPS relies on survey responses and therefore could be subject to recall bias. However, MEPS charge information is based on responses from both provider and patient, and therefore charge variations between diseases should not be affected.

Second, diagnoses were reported using the Clinical Classification Software (CCS). While we did describe patient-level clinical comorbidities present in the data, we did not investigate how variation in charges could be due to differences in patient condition severity or other factors unable to be captured from these administrative datasets. For instance, the diagnosis “normal pregnancy and delivery” included a wide spectrum of presentations ranging from woman in active labor needing admission to otherwise healthy woman during the course of their pregnancy. Further research should try to elucidate the variation in costs controlling for such clinical severity factors.

Finally, we did not adjust for the facility where treatment was received. Differences in baseline charges between hospitals have been well documented and are in part due to factors including geographical differences, provider reimbursement variation, and health care monopolies. [23], [24], [25] In addition, facilities differ in the level of services they provide. A person with a headache at one facility may not receive imaging, for example, whereas a person treated at another facility may receive a head CT. Our intention, however, was not to delineate these differences, given patients presenting to the ED will not be able to predict the services they require. Thus we are trying to describe the patient experience rather than find the source of variability. However, further research should look at the differences between and within hospitals regarding the charge variation for specific diagnoses and procedures to examine more concretely how cost-control measures could work to address any inefficiencies.

### Conclusion

Emergency departments play a valuable role in healthcare delivery, yet consumers know little concerning their ED charges before they receive the bill. In this context, we have identified a high charge burden and charge variation for those that seek outpatient care in the ED. Whether or not acute care charge transparency will aid in mitigating costs still needs to be investigated, however, better information for patients and providers on consumer cost of medical care going forward will allow patients to be aware of the charges they face in the ED.
